# Incident light and morphology determine coral productivity along a shallow to mesophotic depth gradient

**DOI:** 10.1002/ece3.8066

**Published:** 2021-08-30

**Authors:** Michael P. Lesser, Marc Slattery, Curtis D. Mobley

**Affiliations:** ^1^ Department of Molecular, Cellular and Biomedical Sciences, and School of Marine Science and Ocean Engineering University of New Hampshire Durham NH USA; ^2^ Department of BioMolecular Sciences University of Mississippi Oxford MS USA; ^3^ Sammamish WA USA

**Keywords:** coral morphology, mesophotic coral reefs, optics, productivity, topography

## Abstract

1. While the effects of irradiance on coral productivity are well known, corals along a shallow to mesophotic depth gradient (10–100 m) experience incident irradiances determined by the optical properties of the water column, coral morphology, and reef topography.
2. Modeling of productivity (i.e., carbon fixation) using empirical data shows that hemispherical colonies photosynthetically fix significantly greater amounts of carbon across all depths, and throughout the day, compared with plating and branching morphologies. In addition, topography (i.e., substrate angle) further influences the rate of productivity of corals but does not change the hierarchy of coral morphologies relative to productivity.
3. The differences in primary productivity for different coral morphologies are not, however, entirely consistent with the known ecological distributions of these coral morphotypes in the mesophotic zone as plating corals often become the dominant morphotype with increasing depth.
4. Other colony‐specific features such as skeletal scattering of light, Symbiodiniaceae species, package effect, or tissue thickness contribute to the variability in the ecological distributions of morphotypes over the depth gradient and are captured in the metric known as the minimum quantum requirements.
5. Coral morphology is a strong proximate cause for the observed differences in productivity, with secondary effects of reef topography on incident irradiances, and subsequently the community structure of mesophotic corals.

## Graphical abstract

Corals along a shallow to mesophotic depth gradient (10–100 m) experience incident irradiances determined by their morphology and reef topography. Both the substrate angle (i.e., topography) and a hierarchy of colony morphologies have significant, and repeatable, effects on modeled productivity in mesophotic corals.

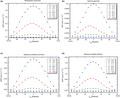

## 1 INTRODUCTION

Operating on ecological timescales, mesophotic coral reef ecosystems (MCEs) are deep (30–150 m) communities structured primarily by strong gradients of light and trophic resources (Laverick et al., 2020; Lesser et al., 2009, 2018, 2019). However, over longer evolutionary timescales, the influence of geological history and geomorphology on modern coral reefs are critical factors that determine their topography, which translates into differences in community composition and function (Locker et al., 2010; Sherman et al., 2010). The reef‐to‐reef differences in community composition associated with differences in topography are best explained by changes in incident irradiances on the reef (Lesser et al., 2018, 2021). While upper MCE (30–60 m) communities are considered a transitional fauna, lower MCE (>60 m) communities are composed of numerous deep reef specialists including scleractinian corals, sclerosponges, and demosponges, as well as soft corals, not found in upper MCEs or shallower waters (Bridge et al., 2012; Laverick et al., 2020; Lesser et al., 2018, 2019; Macartney et al., 2020; Slattery & Lesser, 2012, 2021).

The underwater light environment significantly influences the photobiology of shallow tropical reef corals (Chalker et al., 1988; Falkowski et al., 1990; Gattuso et al., 2006), as well as the ecological structure of both shallow and MCE communities (Kahng et al., 2010; Laverick et al., 2020; Lesser et al., 2009, 2018, 2019). While the optical characteristics of the water column have been described using diffuse attenuation coefficients (K_dPAR_ m^−1^) calculated from downwelling irradiances (E_d_) over the photosynthetically active radiation (PAR) wavelengths from 400 to 700 nm (Hochberg et al., 2020; Kirk, 1994; Mobley, 1994), the interaction of E_d_ with the benthos, and whether the substrate is horizontal, sloping, or vertical in nature affect the incident light that individual corals actually “see” (Brakel, 1979; Lesser et al., 2018, 2021). Recent optical modeling approaches have clearly shown that both substrate slope and colony morphology effect the amount of incident light incident upon a coral colony (Lesser et al., 2018, 2021) and that E_d_, typically measured for the water column on coral reefs, does not adequately describe the incident irradiance on individual colonies (Lesser et al., 2021). In fact, the amount of PAR available for photoautotrophic organisms is significantly decreased, relative to open water measurements of E_d_, by as much as 60%–70% on a vertical wall at midday depending on depth (Lesser et al., 2018, 2021).

The ability of scleractinian corals and their endosymbiotic dinoflagellates (Symbiodiniaceae) to photoacclimatize is a function of their ability to regulate the photosynthetic apparatus under varying irradiances (Dubinsky et al., 1984; Iglesias‐Prieto et al., 2004; Lesser et al., 2000; Mass et al., 2010; Stambler & Dubinsky, 2005; Warner & Suggett, 2016; Wyman et al., 1987). The most studied aspect of the photobiology of scleractinian corals is the variability in the species of Symbiodiniaceae harbored by different corals (Lajeunesse et al., 2018), and how these different species may facilitate survival during exposure to climate change (Suggett et al., 2017). Additionally, Symbiodiniaceae genotypes also change in corals with increasing depth into the lower MCE (Bongaerts et al., 2015; Einbinder et al., 2016; Lesser et al., 2010; Padilla‐Gamiño et al., 2019). Given the effects of reef topography and morphology on incident irradiances at the level of the colony (Lesser et al., 2021), how does this translate into differences in primary productivity? Here, modeled outputs of instantaneous and daily integrated irradiances incident upon mounding, plating and branching corals from different reef topographies (Lesser et al., 2021) are used to calculate rates of productivity (i.e., daily integrated carbon fixation) for corals on a horizontal back reef, a sloping fringing reef, and a vertical forereef over a shallow to mesophotic gradient. Additionally, the efficiency of light utilization for photosynthesis (i.e., minimum quantum requirements [1/ϕ_m_]) was examined to provide additional information for each coral on the efficiency of using the absorbed incident irradiances for photosynthesis. These estimates of depth‐dependent carbon fixation show that daily integrated coral productivity is influenced significantly by both reef topography and coral morphology. The differences between corals in integrated productivity are reflected in their respective 1/ϕ_m_ values and how it changes with depth. Both productivity and 1/ϕ_m_ are light‐dependent emergent features of these corals with their respective morphologies and should be considered as proximate causes for the observed changes in the abundance and distribution of corals from shallow to mesophotic depths.

## 2 MATERIALS AND METHODS

### 2.1 Productivity and minimum quantum requirements

For the productivity calculations, the irradiance data from Lesser et al. (2021), which modeled PAR irradiances denoted as PAR_hs_, PAR_cos_, and PAR_br_ simulating the hemispherical or mounding, plating, and branching morphologies of scleractinian corals, respectively, from shallow to mesophotic depths (i.e., 10, 12, 20, 30, 40, 50, 60, 65, 75, and 100 m) were used. Specifically, hemispherical scalar irradiance, PAR_hs_, is the light incident on an isolated mounding coral per unit area of the reef surface it occupies, while planar irradiance, PAR_cos_, is the light intercepted per unit area for a plating coral where the plates are orientated parallel with the reef surface, and PAR_br_ is the average light incident per unit area of the coral surface for branching corals (Lesser et al., 2021).

Data for 1/ϕ_m_ and maximum gross primary productivity (GPP) were obtained from studies on corals from shallow to mesophotic depths with morphologies (=sensors as a proxy) representative of those simulated above in the radiative transfer model for incident PAR. For the hemispherical sensor, data from the mounding coral, *Montastraea cavernosa*, were used (Lesser et al., 2010; Wyman et al., 1987). For the cosine sensor, data from the plating coral, *Agaricia agaricites*, from Wyman et al. (1987) were used. For the branching morphology, the productivity data, in both summer and winter, from the branching coral, *Stylophora pistillata*, were used (Mass et al., 2007). All empirical productivity data, except for Lesser et al. (2010) that were derived using an optical approach (sensu Hochberg & Atkinson, 2008), were directly measured as in situ oxygen fluxes, and in all cases, the irradiances were originally measured as quantum scalar irradiances. All data were normalized to surface area, and where these rates were initially normalized to a different parameter (e.g., chlorophyll), they were converted to surface area using measurements of colony chlorophyll and surface area reported in the respective publications.

The rates (i.e., µmol O_2_ m^−2^ hr^−1^) of GPP for each coral morphology from each depth were then converted to daily productivity data based on a 12‐hr L:D cycle (i.e., mol O_2_ m^−2^ d^−1^) as were the depth‐dependent irradiances (µmol quanta m^−2^ hr^−1^) from the model runs (Lesser et al., 2021) converted to total daily irradiances (i.e., mol quanta m^−2^ d^−1^). The productivity data were then converted into units of carbon (i.e., mol C m^−2^ d^−1^) using depth‐specific photosynthetic quotient (PQ) values from a regression of the irradiance‐dependent changes in PQ (Gattuso & Jaubert, 1990) as previously described (Lesser, 2013).

Depth‐dependent changes in GPP based on carbon were calculated as a function of solar zenith angle (*θ*_sun_) from sunrise to sunset for a shallow to mesophotic depth gradient that includes a vertical wall beginning at ~60 m. Then, the total daily irradiances (i.e., mol quanta m^−2^ d^−1^) at each depth were regressed against the daily integrated GPP. The changes in integrated GPP with integrated irradiances were also calculated for corals at 65, 75, and ~100 m for a sloping topography starting at 60 m, based on the irradiance model outputs (Lesser et al., 2021), and compared with the same depths from a vertical wall using an analysis of covariance (ANCOVA) to assess the difference in productivity between species, with depth as the covariate. All data were assessed for normality and heteroskedasticity before analysis and, where needed, transformed for analysis and back‐transformed for presentation.

Using the data sources described above, the minimum quantum requirements (i.e., 1/ϕ_m_) for each coral species, representing different morphologies, were determined for the same depths described in the productivity calculations by using irradiances calculated for the two different reef topographies (i.e., slope‐to‐slope and slope‐to‐wall; Lesser et al., 2021). The 1/ϕ_m_ values were then regressed as a function of depth using a power function as it resulted in the best fits (i.e., highest *R* ^2^ values).

## 3 RESULTS

**FIGURE 1:**
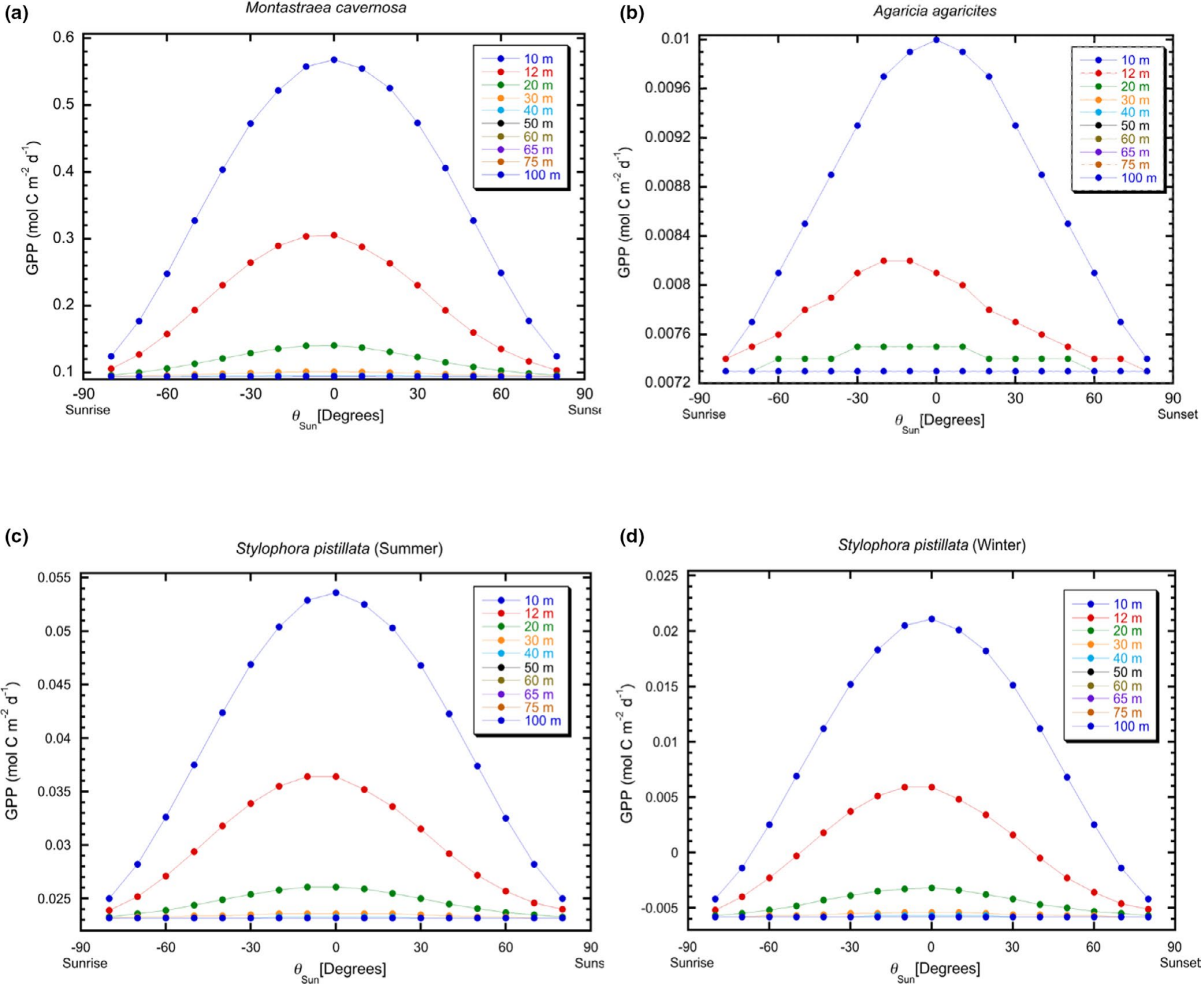
(a‐d) Depth‐dependent changes in gross primary production (GPP) based on modeled irradiances for each coral species and depth (10, 12, 20, 30, 40, 50, 60, 65, 75, and 100 m). Points for each depth are GPP as a function of sun angle. (a) GPP for hemispherical or mounding coral (*Montastraea cavernosa*) as a function of depth. (b) GPP for a cosine or plating coral (*Agaricia agaricites*) as a function of depth. (c) GPP for branching (*Stylophora pistillata*) coral in summer as a function of depth and (d) GPP for branching (*Stylophora pistillata*) coral in winter as a function of depth. Calculations assume 100% absorption of all available quanta as *θ*_Sun_ traverses the sky from sunrise to sunset

At each depth, the orientation to the sun for each coral is determined by reef topography, and it is assumed that 100% of the fraction of the incident irradiance available after interacting with a spectrally resolved coral reflectance (Lesser et al., 2021), regardless of its morphology, is absorbed and used for photochemistry. For all corals and depths, the GPP for each coral morphology was seen to vary over the day, and with depth, in a qualitatively similar manner based on solar zenith angle (Figure 1a–d). The magnitudes of the differences (note scaling of y‐axes), however, depend strongly on the morphology of the coral (Figure 1a–d).

**FIGURE 2:**
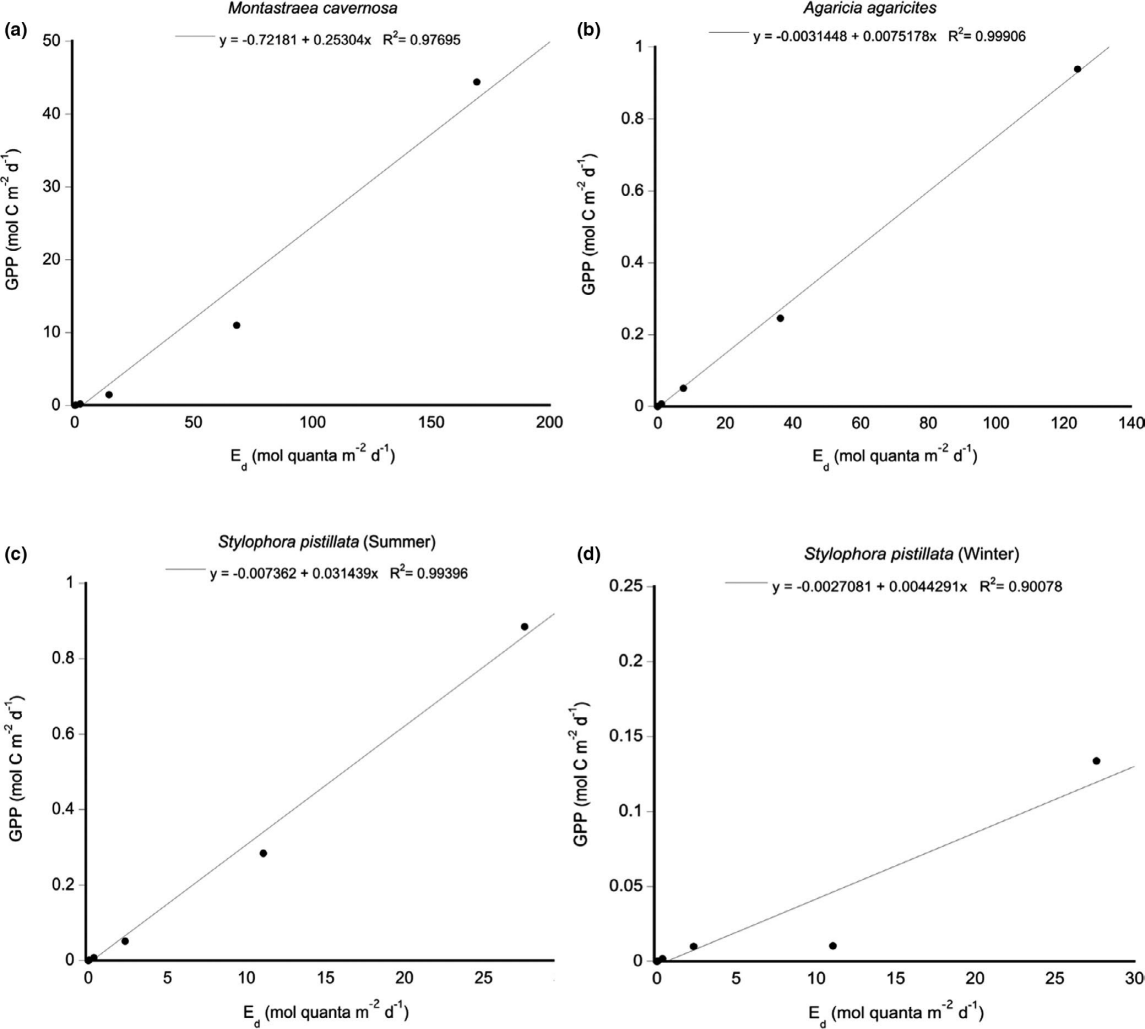
Linear regression of productivity potential versus depth (10, 12, 20, 30, 40, 50, 60, 65, 75, and 100 m) for coral species representing (a) hemispherical or mounding coral (*Montastraea cavernosa*), (b) cosine or plating coral (*Agaricia agaricites*), (c) branching coral (*Stylophora pistillata*) in summer and (d) branching coral (*Stylophora pistillata*) in winter. All regressions were highly significant (ANOVA: *p* < .05)

Given that coral GPP integrated over the day has been shown to be linearly related to irradiances integrated over the day (Sawall & Hochberg, 2018), we regressed the daily integrated irradiances from the model against the calculated daily integrated GPP in units of carbon. Using the integrated daily PAR values (mol quanta m^−2^ d^−1^), the change in daily integrated GPP (mol C m^−2^ d^−1^) for the modeled runs that included a vertical wall showed a linear increase with increasing irradiance for all coral morphologies (*R* ^2^ from 0.90 to 0.99: Figure 2a–d). When we integrate GPP across depths for a reef topography with a vertical wall, *Montastraea cavernosa* colonies had a daily GPP potential of 115.17 mol C m^−2^ d^−1^ that was positive at all depths, and *Agaricia agaracites* colonies had a GPP potential of 2.72 mol C m^−2^ d^−1^ that was also positive at all depths. Colonies of *Stylophora pistillata* in the summer showed a GPP rate of 2.86 mol C m^−2^ d^−1^ with positive rates of GPP at all depths throughout the day, while in the winter, colonies of *S. pistillata* showed a GPP rate of 5.82 x 10^–7^ mol C m^−2^ d^−1^ that only exhibited positive rates at 10 m and 12 m. Doing the same for the sloping topography revealed an increase of ~3% in integrated GPP for colonies of *M. cavernosa* (118.77 mol C m^−2^ d^−1^), ~72% for *A. agaricites* (4.67 mol C m^−2^ d^−1^), ~19% for *S. pistillata* in summer (3.41 mol C m^−2^ d^−1^), and ~1% for *S. pistillata* in winter (3.88 × 10^–5^ mol C m^−2^ d^−1^) where positive rates of GPP occurred at 10 m, 12 m, and 20 m.

Differences in GPP based on differences in topography (vertical versus sloping substrate) at 65, 75, and 100 m were assessed using ANCOVA, where all assumptions of ANCOVA were satisfied (e.g., homogeneous slopes) for all comparisons. There was no significant increase in GPP across those lower mesophotic depths for *M. cavernosa* colonies (ANCOVA: *F*_1,2_ = 11.16, *p* = .079) or *S. pistillata* colonies in winter (ANCOVA: *F*_1,2_ = 4.66, *p* = .164), but there were significant increases in GPP for both colonies of *S. pistillata* in summer (ANCOVA: *F*_1,2_ = 18.77, *p* = .049) and colonies of *A. agaricites* (ANCOVA: *F*_1,2_ = 27.89, *p* = .034) on sloping substrates.

**FIGURE 3:**
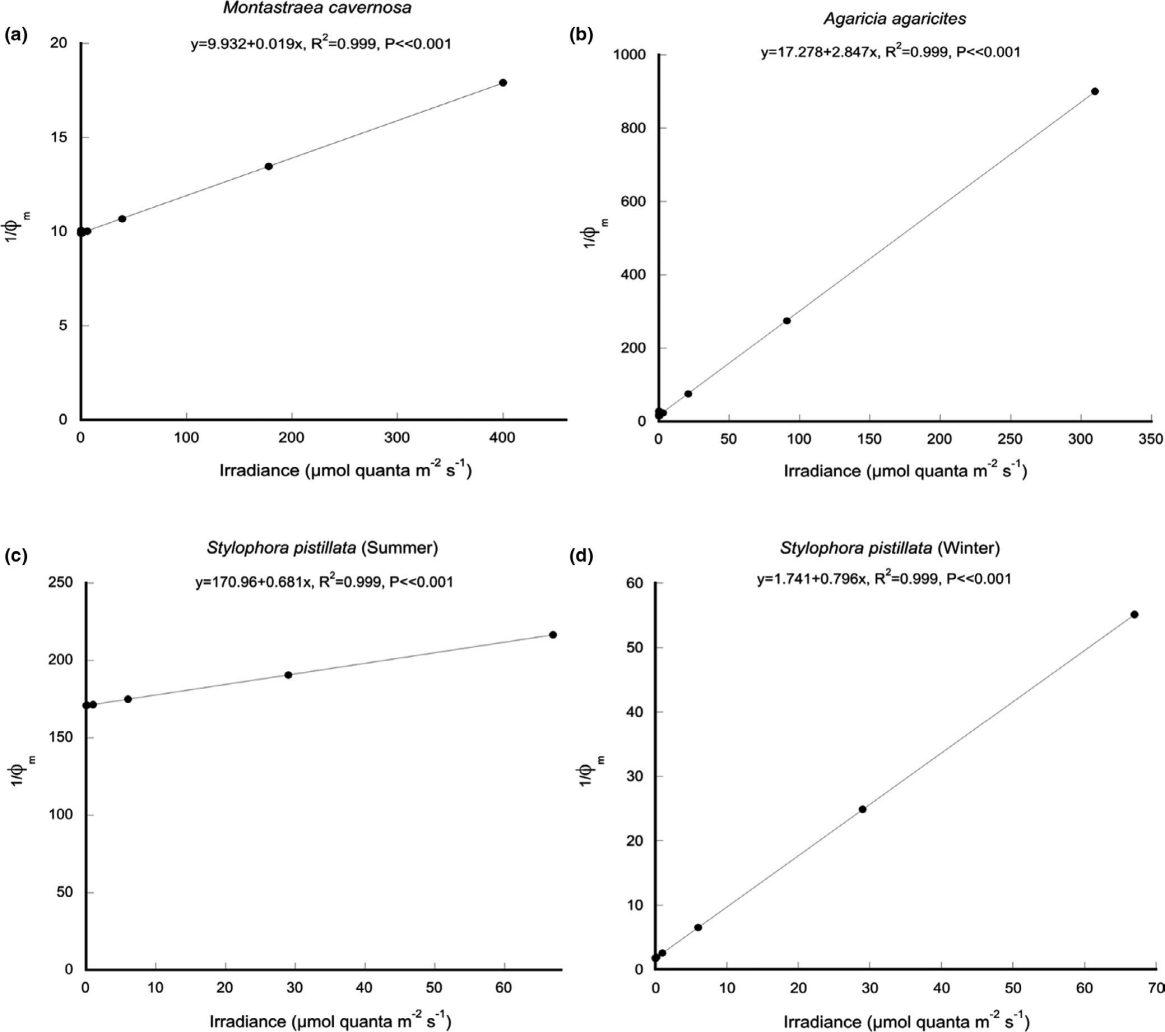
Linear regression of minimum quantum requirements (1/ϕ_m_) versus instantaneous irradiance of photosynthetically active radiation for coral species representing (a) hemispherical or mounding coral (*Montastraea cavernosa*), (b) cosine or plating coral (*Agaricia agaricites*), (c) branching coral (*Stylophora pistillata*) in summer, and (d) branching coral (*Stylophora pistillata*) in winter. All regressions were highly significant (ANOVA: *p* < .05)

The relationship between instantaneous PAR (µmol quanta m^−2^ s^−1^), and the minimum quantum requirements for photosynthesis (1/ϕ_m_) showed a highly significant linear increase (ANOVA: *F*_1,9_, *p* « .0001) with increasing irradiance for all species (*R* ^2^ > 0.99: Figure 3a‐d). For each species, a slope‐to‐wall versus a slope‐to‐slope topography comparison of 1/ϕ_m_ at 65 m, 75 m, and 100 m depths was assessed using ANCOVA where all assumptions were satisfied for all comparisons. There was no significant change in 1/ϕ_m_ on sloping versus vertical substrates for colonies of *M. cavernosa* (ANCOVA: *F*_1,2_ = 4.61, *p* = .165), *A. agaricites* (ANCOVA: *F*_1,2_ = 4.47, *p* = .169), *S. pistillata* in summer (ANCOVA: *F*_1,2_ = 4.52, *p* = .167), and *S. pistillata* in winter (ANCOVA: *F*_1,2_ = 0.001, *p* = .999).

## 4 DISCUSSION

In the wider Caribbean basin, the Red Sea, and the Indo‐Pacific, the distribution of corals shows a general decline in percent cover with depth (but see Kramer et al., 2020, for an exception in the Red Sea). Within this consistent decline in scleractinian corals with increasing depth are breaks in coral communities between the upper and lower mesophotic zones at ~50‐ to 60‐m depth (Liddell et al., 1997; Liddell & Ohlhorst, 1987, 1988), which represent a repeatable response to reduced irradiance at depth for mesophotic reefs worldwide (Lesser et al., 2021; Lesser et al., 2019; Tamir et al., 2019). Embedded in this general pattern, we also see that coral morphotypes vary across depth gradients with greater abundances of platelike corals at mesophotic depths, followed by mounding morphs, and the near absence of branching coral species (Bridge et al., 2012; Fricke & Meischner, 1985; Goreau & Goreau, 1973; Goreau & Wells, 1967; Hoeksema et al., 2017; Kahng et al., 2010; Kramer et al., 2020; Kühlman, 1983; Liddell et al., 1997; Liddell & Ohlhorst, 1987, 1988).

The results presented here show that both coral morphology and reef topography significantly influence the rates of daily integrated GPP in scleractinian corals as a function of depth. A caveat to consider is the modeled irradiances are planar in nature, while all the empirical studies of coral productivity used here measured E_d_ as quantum scalar irradiances (Lesser et al., 2010; Mass et al., 2007; Wyman et al., 1987). As a result, the actual irradiances used to estimate GPP in these corals could be lower by as much as a factor of four, while the differences between species persist. No quantum scalar irradiance meters, however, are truly 4π as there is a large blind spot for the connector. Additionally, sensor placement in the empirical studies for coral productivity involved a significant amount of shading of upwelling (E_u_) irradiances making these measurements functionally more like 2π sensors (i.e., planar).

On coral reefs, light is an important abiotic factor that regulates rates of productivity, calcification, and growth (e.g., Mass et al., 2007). Previous simulations of the underwater light environment from shallow to MCE depths show that downwelling planar irradiance, E_d_, measured for the water column is not what corals on substrates of varying angle (i.e., horizontal versus vertical versus sloping) are seeing (Lesser et al., 2018, 2021). Thus, reef topography can have significant effects on coral productivity. Additionally, coral morphology adds another factor with mounding corals having better bio‐optical characteristics for capturing light per unit reef area (Lesser et al., 2021), and within colony endosymbiotic populations that are genetically different and acclimatized to the coral's architecture‐mediated light quality and quantity (Rowan & Knowlton, 1995). These bio‐optical characteristics at the colony level result in greater depth integrated GPP at all sun and substrate angles for mounding colonies compared with plating corals per unit area. Branching coral colonies represent a continuation of this morphological gradient in GPP; they have more surface area for the same area of reef, but the efficiency of light capture decreases more than the other morphologies because of self‐shading (Kaniewska et al., 2014; Lesser et al., 2021), as does productivity.

If we look specifically at substrate angle, by extending our 60° fringing reef slope onto the fore reef out to a depth of ~100 m (i.e., slope‐to‐slope topography) an increase in GPP occurs in all coral morphologies at 65, 75, and ~100 m (Appendix S1), compared with the same species on a slope‐to‐wall topography, as a result of the increase in incident irradiances on sloping substrates relative to vertical walls (Lesser et al., 2021). In this study, a hierarchy of coral morphology from mounding to plating to branching corals showed a decrease in GPP, regardless of reef topography although reef topography had additional secondary effects. A large, and significant, increase in GPP occurred in *Agaricia agaricites*, a plating coral, while the smallest increase occurred in *Stylophora pistillata*, a branching coral, on sloping versus vertical topography. This species‐specific variability illustrates that there is a limit to how much changes in coral morphology affect rates of GPP and calcification, which have been shown to decline significantly with increasing depth into the mesophotic zone (Lesser et al., 2010; Mass et al., 2007).

Looking at the differences in depth integrated GPP between coral morphologies, mounding corals outperform all other morphologies, at all depths. But these results do not explain the observed phenotypic plasticity changes in morphology from hemispherical to plating morphology with increasing depth on many coral reefs (e.g., Lesser et al., 2010), or the phenotypic plasticity observed in agaricids with changing irradiances (Anthony et al., 2005; Hoogenboom et al., 2008). Additionally, a significant number of coral species with platelike morphologies exhibit extreme endemism in the lower mesophotic (e.g., Padilla‐Gamiño et al., 2019). Another metric that captures photosynthetic performance under a range of irradiances is the minimum quantum requirements for photosynthesis, or 1/ϕ_m_. This metric tells us how many quanta are required to produce a molecule of oxygen and encapsulates the effects of tissue absorption, scattering by the skeleton, the package effect, and ultimately absorption by the photosynthetic pigments of the endosymbiotic Symbiodiniaceae (Brodersen et al., 2014; Dubinsky et al., 1984). The data required to calculate 1/ϕ_m_ are unavailable for most species of coral, especially along the entire shallow to lower mesophotic depth range. However, data from Lesser et al. (2010) for *Montastraea cavernosa* showed that 1/ϕ_m_ declined with increasing depth, as it does for all corals in this study. As discussed in Wyman et al. (1987), multiple coral species from the Caribbean, at increasingly lower irradiances, became more efficient at using the absorbed quanta. In addition, the colony morphology of *M. cavernosa* began to change its shape from mounding to plating at ~45–61 m suggesting added benefits to the flattened (i.e., cosine) morphology under low irradiances (Lesser et al., 2010). Based on the highest variability explained (i.e., *R* ^2^) if you fit a power function to 1/ϕ_m_ versus depth (Appendix S2), and solve for the theoretical minimum quantum requirements of 8 quanta O_2_^−1^, the maximum depth where photosynthesis could occur, regardless of the actual depth distribution, for *M. cavernosa* is ~165 m, for *A. agaricites* ~99 m, for *S. pistillata* (winter) ~22 m, and for *S. pistillata* (summer) >200 m.

The differences in productivity between coral morphologies reported here do not consider the effects of depth‐dependent changes, and endemism, known to occur in the community composition of the photoautotrophic endosymbiont (Symbiodiniaceae) of corals found along the shallow to mesophotic light gradient (Bongaerts et al., 2015; Lesser et al., 2010; Pochon et al., 2015; Ziegler et al., 2015). Additionally, several of these identified Symbiodiniaceae, primarily in the genus *Cladosporium* sp., are unique and can photoacclimatize a extremely low irradiances (Einbinder et al., 2016; Padilla‐Gamiño et al., 2019). Lastly, the difference in the functional performance of an ideal versus a real sensor is very likely to be a function of the small‐scale optics in the skeleton of different coral morphologies (Enríques et al., 2005, 2017; Kühl et al., 1995; Wangpraseurt et al., 2012). Differences in skeletal architecture can cause varying degrees of scattering, which results in an “amplification” factor that varies with the morphology of the coral (Enríques et al., 2017). In low irradiance environments, and when combined with the unique photosynthetic characteristics of Symbiodiniaceae in mesophotic corals (e.g., Einbinder et al., 2016), the skeletal morphology becomes a significant contributor to the light available for photosynthesis. Using a metric of integrated scattering over the entire coral skeleton called the “light enhancement factor” (LEF), Enríques et al. (2017) showed that plating coral species had greater LEF values, up to twice as high, compared with massive coral species This, along with species‐specific differences in 1/ϕ_m_, which captures differences in skeletal microarchitecture, tissue thickness, and the Symbiodiniaceae communities, contributes to our understanding of how a plating coral such as *Agaricia agaricites* is able to photosynthesize at mesophotic depths, and why a mounding coral such as *Montastraea cavernosa* transitions to a plating morphology as depth increases and irradiance decreases. Branching corals, regardless of season, succeed in shallow reef environments because a limiting resource, light, is available in excess. Branching species, however, are at a distinct disadvantage as depth increases because as the availability of light decreases, the profound self‐shading in branching species results in rates of photosynthesis below the compensation point (Lesser et al., 2021).

Many studies on corals suggest that the attenuation of solar irradiance, and the transition from photoautotrophy to heterotrophy, is the most important factor regulating the observed patterns of zonation from shallow to mesophotic depths (Lesser et al., 2009, 2018, 2019; Martinez et al., 2020). Recent studies have included better spatial and temporal coverage of irradiance measurements to define community transitions along the shallow to mesophotic depth gradient based on the attenuation of light as opposed to depth per se (Laverick et al., 2020; Tamir et al., 2019). Many studies, however, retain current depth definitions for upper and lower mesophotic depth boundaries as a reference point, and do not explicitly examine what individual corals at any depth on a reef actually “see” as it relates to light, and how that might mechanistically relate to function. In the study by Laverick et al. (2019), studying the plating coral, *Agaricia lamarcki*, from shallow (<30 m) to upper mesophotic (30–60 m) depths in the Caribbean, it was suggested that the physiological similarities of coral samples from both shallow reefs and upper mesophotic depths were a function of similar irradiance microhabitats, making depth an inappropriate proxy to ecologically characterize coral populations from discrete depths. For the mounding coral, *Montastraea cavernosa*, Lesser et al. (2010) found that corals from shallow and upper mesophotic depths were also physiologically similar in many respects and that multiple physiological characteristics did not differ significantly in a depth‐dependent manner until the lower mesophotic zone (60–150 m). Contraction of ecological zones or the presence of endemic mesophotic corals in shallow habitats such as lagoons (Colin & Linfield, 2019; Laverick et al., 2020) provides additional evidence for the strong influence of irradiance on the ecological distribution of corals. Here, we have shown that differences in GPP, and by proxy energetic fluxes through individual coral colonies, for different reef topographies and coral morphologies from shallow to mesophotic depths are driven largely by incident irradiances on the colony itself, not the irradiances of the water column. The irradiance model (Lesser et al., 2021) is adaptable to multiple scenarios and can be coupled to other models addressing small‐scale optics and provides an ecological framework for hypothesis testing on the functional attributes of not only corals, but also other photoautotrophs (e.g., macrophytes) on MCEs.

## CONFLICT OF INTEREST

The authors declare no conflicts, or competing, interests.

## AUTHOR CONTRIBUTIONS

**Michael P. Lesser:** Conceptualization (lead); Data curation (supporting); Formal analysis (supporting); Funding acquisition (equal); Investigation (equal); Methodology (supporting); Project administration (lead); Resources (equal); Software (supporting); Supervision (lead); Validation (supporting); Visualization (supporting); Writing‐original draft (lead); Writing‐review & editing (equal). **Marc Slattery:** Conceptualization (equal); Data curation (supporting); Formal analysis (supporting); Funding acquisition (equal); Investigation (equal); Methodology (supporting); Project administration (equal); Resources (equal); Software (supporting); Supervision (equal); Validation (equal); Visualization (equal); Writing‐original draft (supporting); Writing‐review & editing (equal). **Curtis D. Mobley:** Conceptualization (supporting); Data curation (supporting); Formal analysis (equal); Funding acquisition (supporting); Investigation (supporting); Methodology (equal); Project administration (supporting); Resources (supporting); Software (equal); Supervision (supporting); Validation (equal); Visualization (equal); Writing‐original draft (supporting); Writing‐review & editing (equal).

## Supporting information

Appendix S1

Appendix S2

## Data Availability

All irradiance modeling data are available at the Biological & Chemical Oceanography Data Management Office (https://www.bco‐dmo.org/dataset/841083/data).
