# Supplementary material for: Incident light and morphology determine coral productivity along a shallow to mesophotic depth gradient
Source: Ecol Evol. 2021 Aug 30;11(19):13445–54. doi: 10.1002/ece3.8066 (PMC8495790; doi:10.1002/ece3.8066)

Appendix II. Relationship between minimum quantum requirements ($1/\phi_m$) and depth for each coral species using a power function.

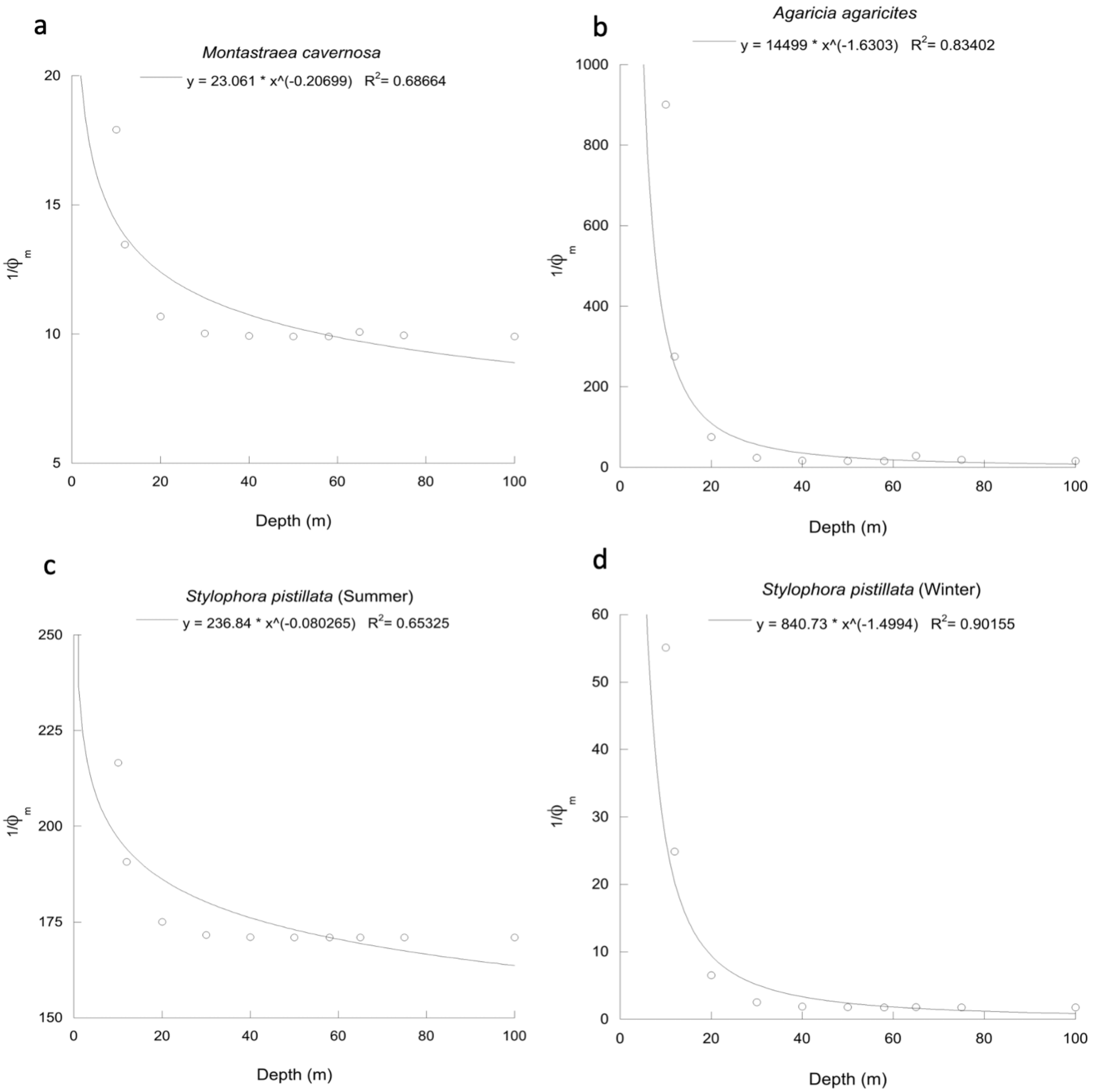

Supplement: Supplementary file 2 — Appendix S2 [file ECE3-11-13445-s002.pdf]
